# Lack of Noradrenergic Modulation of Indirect Semantic Priming

**DOI:** 10.3233/BEN-2009-0236

**Published:** 2009-12-07

**Authors:** Jacquelyne S. Cios, Regan F. Miller, Ashleigh Hillier, Madalina E. Tivarus, David Q. Beversdorf

**Affiliations:** ^1^Department of NeurologyThe Ohio State UniversityColumbusOHUSA; ^2^Department of PsychologyUniversity of Massachusetts-LowellLowellMAUSA; ^3^Department of RadiologyUniversity of RochesterRochesterNYUSA; ^4^Biophysics Graduate ProgramThe Ohio State UniversityColumbusOHUSA; ^5^Departments of RadiologyNeurologyand Psychology and the Thompson CenterUniversity of MissouriColumbiaMOUSA

**Keywords:** semantic, priming, noradrenergic, language, dopaminergic

## Abstract

Norepinephrine and dopamine are both believed to affect signal-to-noise in the cerebral cortex. Dopaminergic agents appear to modulate semantic networks during indirect semantic priming, but do not appear to affect problem solving dependent on access to semantic networks. Noradrenergic agents, though, do affect semantic network dependent problem solving. We wished to examine whether noradrenergic agents affect indirect semantic priming. Subjects attended three sessions: one each after propranolol (40 mg) (noradrenergic antagonist), ephedrine (25 mg) (noradrenergic agonist), and placebo. During each session, closely related, distantly related, and unrelated pairs were presented. Reaction times for a lexical decision task on the target words (second word in the pair) were recorded. No decrease in indirect semantic priming occurred with ephedrine. Furthermore, across all three drugs, a main effect of semantic relatedness was found, but no main effect of drug, and no drug/semantic relatedness interaction effect. These findings suggest that noradrenergic agents, with these drugs and at these doses, do not affect indirect semantic priming with the potency of dopaminergic drugs at the doses previously studied. In the context of this previous work, this suggests that more automatic processes such as priming and more controlled searches of the lexical and semantic networks such as problem solving may be mediated, at least in part, by distinct mechanisms with differing effects of pharmacological modulation.

## 1. Introduction

Groups of representations of words related in meaning are believed to be neurally linked in the semantic network [16,34]. One mechanism of investigating semantic networks is the effect of word association on priming. Priming experiments involve giving subjects a lexical decision task requiring recognition of words (target) after they have been previously exposed to another word (prime). For closely related words such as “black” and “white” (opposites), early studies showed that subjects recognized a word more quickly after previous presentation of a closely related word [32]. The degree of relation between the prime and target words, or semantic distance, has a relationship to the speed of word recognition. For example, whereas subjects exposed to prime words, such as “summer,” react more quickly to closely related words, such as “winter” (direct priming), this effect has been observed to a lesser degree in more distantly related word pairs, such as “summer” and “snow” (indirect priming, since these are only related through a common relation to the

∗Corresponding author: David Beversdorf, MD, University of Missouri, Departments of Radiology, Neurology, and Psychology and the Thompson Center, 300 Portland St, Suite 110, Columbia, MO 65211, USA. Tel.: +1 573 882 6081; Fax: +1 573 884 1151; E-mail: beversdorfd@health.missouri.edu.

ISSN 0953-4180/09/$17.00 © 2009 – IOS Press and the authors. All rights reserved word, “winter”), when compared to pairs of unrelated words [16,25]. Network models propose that this is the result of an increasing degree of overlapping network activation with closer relationships between words [6, 16,30].

The priming effect has been studied by examining varying stimulus onset asynchronies (SOA) as well as varying semantic distance. Increasing the length of time between the exposure to the prime word and the target word, or SOA, increases the indirect priming effect, but not direct priming [41], which may relate to the internodal spread of activation, where two distantly related concepts are more connected in the neural network given sufficient time.

Modulating the semantic network through administration of pharmacological agents has also been attempted. Much research has focused on the dopaminergic system, since it has been shown that dopamine increases strong signals and dampens weak signals in the cortex [10,38]. Kischka et al. have postulated that dopamine increases the signal-to-noise ratio in semantic networks by reducing the spread of activation [25]. They found that oral administration of L-dopa prior to the performance of a word priming task resulted in loss of the indirect priming effect but had no effect on closely related priming. Angwin et al [2] found a loss of both direct and indirect semantic priming with L-dopa at an SOA of 500 ms. Direct and indirect semantic priming were both present in both drug conditions at shorter SOAs (250 ms), and no direct or indirect semantic priming was present in either drug condition at longer SOAs (1200 ms) [2]. More recent evidence suggests that the priming effect is mediated by effects on the D1 dopamine receptor [36,37].

Research has also suggested other neurotransmitter systems as potential modulators of performance on other types of tasks involving the semantic network. Norepinephrine also increases the signal-to-noise ratio in the central nervous system, by selective suppression of excitatory intrinsic connectivity thereby decreasing the background activity of neurons relative to the activity of neurons receiving direct afferent input in brain slice preparations [24]. This is proposed as a mechanism for the superior performance on attentional tasks in the setting of increased norepinephrine [24], and may also relate to impairments in performance on other types of tasks with increased norepinephrine activity believed to be due to decreased intrinsic associative activity in access to networks such as the semantic network [1,9]. Previous research has shown that stress, which increases noradrenergic activity [26,45], has a negative effect on verbal problem solving [1]. Beversdorf et al. showed that performance on anagram- solving tasks, involving access to the lexical/semantic network, is modulated by administration of noradrenergic agents [7,8].

These findings reveal an effect of the noradrenergic system on semantic problem solving tasks, while the dopaminergic system affects the semantic network in priming. Our subsequent research has suggested that dopaminergic agents do not affect performance on semantic network dependent problem solving tasks [40]. The question remains as to whether noradrenergic agents affect the semantic network in priming. Furthermore, L-dopa, used in many of the dopaminergic priming studies, is converted to norepinephrine and dopamine within the central nervous system. After absorption from the gastrointestinal tract and conversion by dopamine decarboxylase to dopamine, a peak concentration is detected by one hour after administration [25,33,35]. The dopamine is also converted by dopamine beta-hydroxylase to norepinephrine. This is well documented in the peripheral tissues [19], but previous studies have also shown increased central nervous system norepinephrine in a rodent model within 50 minutes of systemic administration [11].

Therefore, the aim of the present research is to determine whether the previously reported effects of dopamine on semantic processing might also be found with noradrenergic agents, using the longer SOAs previously shown to exhibit dopaminergic effects on indirect priming [25]. Ephedrine increases central noradrenergic transmission, Therefore, if the noradrenergic system affects semantic priming, ephedrine would restrict the spread of activation of the semantic network and decrease the indirect priming effect. This is the primary hypothesis to be tested. Conversely, propranolol, which decreases the postsynaptic effect of norepinephrine, would be expected to increase the spread of activation of the semantic network, potentially making the indirect priming effect more pronounced. However, if there is no noradrenergic effect on semantic priming, then no effect would be observed with these agents on semantic priming.

## 2. Materials and methods

### 2.1. Subjects

The study protocol was approved by The Ohio State University Office of Research Risks Protection. All subjects provided informed consent before their participation in this study. Subjects were 9 female and 9 male native English speakers aged 20–30, with no previous history of depression, asthma, cardiac or thyroid disease, or recent usage of medications affecting the noradrenergic system. In addition they were instructed not to drink caffeinated beverages on the day of the testing. None had any known language or attention deficit disorders that might affect the results. Based on previous results demonstrating a significant decrease in indirect semantic priming with L-DOPA [25], if ephedrine were to have the same effect as L-DOPA, the power of a study at the level of $\alpha = 0.05$ with 18 subjects would be 0.8 for detecting a significant effect on indirect semantic priming. This sample size is identical to the sample size used in previous studies of the effect of noradrenergic agents on lexical/semantic problem solving using a similar design [7,8], for proper counterbalancing, and is greater than the sample size used in each group in previous work demonstrating the effect of dopaminergic agents on semantic priming using a between-subject design [25].

### *2.2. Materials*

All stimuli were presented with SuperLab software, which presented stimuli and measured response times. Responses were made on a Cedrus Lumina touch-button hand-controlled response pad.

### *2.3. Procedures*

A double-blinded, placebo-controlled, crossover within subject design was employed. Subjects attended 3 sessions each, one week apart at the same time of day, and received three separate sets of priming stimuli, arranged such that the order in which subjects were exposed to word pairs of varying degrees of semantic distance was counterbalanced. The order in which the subjects received noradrenergic stimulant (ephedrine 25 mg administered orally), noradrenergic antagonist (propranolol 40 mg administered orally), or placebo was also counterbalanced. As the administration was double blinded, subjects were not warned to expect any particular drug effect before administration of each drug. Forty-five minutes were allowed between administration of the drug and the start of the lexical decision task, as with our previous work on verbal problem solving [7]. Heart rate and blood pressure were obtained immediately before administration of the drugs and after the computer word test to assess the degree of hemodynamic effect of the drugs.

### *2.4. Stimuli*

The first word, or prime, appeared on the screen for 500 msec, which was followed by a "+" sign for the period of 500 msec, and then the target word appeared for 1000 msec or until a response was made, followed by a blank screen for 2000 msec. The subjects were given a total of 3000 msec to respond by pushing the button as to whether or not the second word of the pair is a word or a non-word. If the subject did not respond by the end of this designated period of time, the program did not record a response to that item, and, as with incorrect responses, these were not included in the analysis, but their frequency was noted. At each session, one hundred word pairs were presented with 20 closely, 20 distantly, and 40 unrelated word pairs, and 20 pairs where the second word was a non-word. The words were from our previous imaging work [43], and were categorized as defined by Kischka et al. [25], where closely related words were one associative step apart (summer-winter), distantly related words were two associative steps apart (summer-snow, the connection between the prime and target is obvious only through a mediating associated word, in this case winter), and unrelated words had no association (ladder-computer). The lists were tested on other volunteers prior to the experiment to confirm the expected differential reaction times between categories. All words and non-words were between 3 and 7 letters, with an average length of 5.06 letters, and there were no significant differences between the three word pair conditions for written word frequency, concreteness, or imageability (MRC Psycholinguistic Database, http://www.Psy.uwa.edu.au/mrcdatabase/uwa_mrc.htm).

*Analysis*: The response times for correct responses indicating recognition of the target as a real word in the English language were recorded onto a spreadsheet. Trials with no response and incorrect responses were tallied separately. The correct affirmative and negative responses were averaged for each condition for each subject (adjusted for test order for learning effect and test version for variability in task difficulty), and compared within subject between the three drug conditions (propranolol, ephedrine, and placebo) and semantic distance conditions (closely related, distantly related, and unrelated) within subject, using repeated measures analysis of variance (ANOVA), as well as specific examination of the priming effects with placebo and ephedrine to test the primary hypothesis. Heart rates and blood pressures were also compared within subject between drug conditions.

Table 1
Comparisons of the priming effect between the three levels of semantic distance for ephedrine and placebo: Statistical comparison between semantic distances in response times under the condition of ephedrine (top), placebo (bottom). RT= reaction time in seconds.

| **Ephedrine** | **Mean RT** (**Std Dev**) | **t(17)** = | **p=** |
|---|---|---|---|
| Closely | 505 (58) | | |
| Distantly | 509 (70) | | |
| Unrelated | 539 (70) | | |
| Closely vs. Distantly | | 0.62 | 0.75 |
| Closely vs. Unrelated | | 0.37 | 0.002 |
| Distantly vs. Unrelated | | 0.34 | 0.003 |
| **Placebo** | **Mean** (**Std Dev**) | **t(17)** = | **p=** |
| Closely | 528 (83) | | |
| Distantly | 524 (94) | | |
| Unrelated | 546 (83) | | |
| Closely vs. Distantly | | 0.45 | 0.66 |
| Closely vs. Unrelated | | 4.20 | 0.001 |
| Distantly vs. Unrelated | | 3.12 | 0.006 |

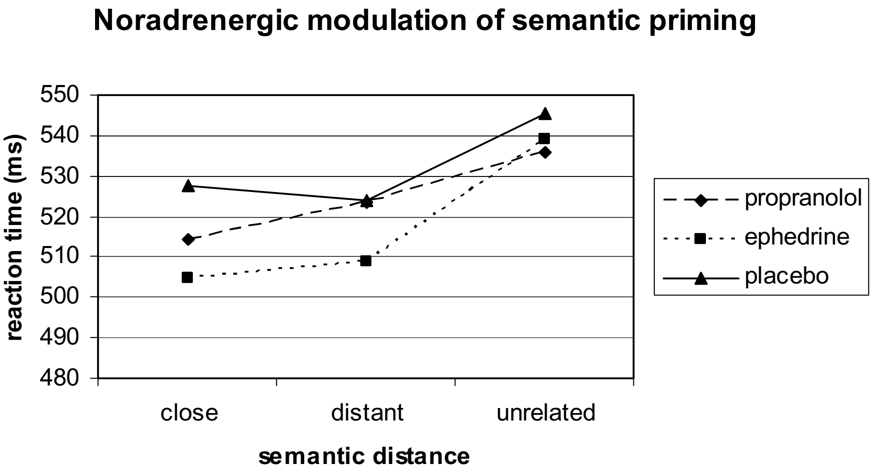

Fig. 1. Average response latency to the target word on the lexical decision task for each drug and each semantic distance of the word pairs.

## 3. Results

As expected, heart rate (HR) and systolic blood pressure (SBP) tended to be lower after administration of propranolol than after placebo (for HR, 68.7 (sdev 9.1) vs. 73.3 (sdev 9.7), $p = 0.05$; for SBP, 112.7 (sdev 13.9) vs. 118 (sdev 12.8), $p = 0.08$). Differences did not reach significance in HR or blood pressure after ephedrine vs. after placebo. In the comparison of pre-drug vs. post-drug, significant decreases were observed for HR, SBP and DBP after propranolol (for HR, 77.4 (sdev 13.6) vs. 68.7 (sdev 9.1), $p = 0.007$; for SBP, 122.3 (sdev 12.5) vs. 112.7 (sdev 13.9), $p = 0.005$; for DBP, 78.6 (sdev 9.2) vs. 74.6 (sdev 8.7), $p = 0.02$). Significant increases were not detected for SBP, DBP or HR for ephedrine, and no pre-drug vs. post-drug changes were seen with any of the measures with placebo.

In order to examine the effect of ephedrine on semantic priming, initial ANOVAs were performed to compare response times between the three relatedness conditions. Ephedrine and placebo showed significant differences between the three levels (F(2,34) = 6.299, $p = 0.005$ for ephedrine; F(2,34) = 5.830, $p = 0.007$ for placebo) (Fig. 1). Post-hoc within subject t-tests for both ephedrine and placebo show significant differences between closely vs. unrelated, and distantly vs. unrelated, but not between closely related vs. distantly related. Therefore, a priming effect was shown for ephedrine and placebo conditions for closely and distantly related words as compared to unrelated word pairs. (Table 1).

A 3×3 repeated measures ANOVA comparing reaction time between repeated factors of semantic relatedness and drug (closely vs. distantly vs. unrelated x ephedrine vs. placebo vs. propranolol) revealed significant main effect of relatedness (F(2,16) $=$ 38.5, P$<$ 0.0005), but no main effect of drug (F(2,16) $=$ 1.65, $P = 0.223$) or drug - relatedness interaction (F(6,16) $=$ 0.76, $P = 0.57$). In addition, repeated measures ANOVA comparing drug conditions within each semantic distance revealed no significant drug effects for closely related pairs (F(2,34) $=$ 1.464, $P = 0.079$), distantly related (F(2,34) $=$ 0.408, $P = 0.668$), and unrelated (F(2,34) $=$ 0.241, $P = 0.787$). Post-hoc related t-tests were conducted comparing the three drugs for the trend observed for closely related pairs. Only a trend was found for a faster response on ephedrine compared to placebo for closely related pairs (t(17) $=$ 1.925, $p = 0.071$). Numbers of errors and non-responses were less than 5% for each drug condition.

## 4. Discussion

Previous research had shown an effect of dopamine inhibiting the indirect semantic priming effect on word recognition tasks by restricting the semantic network [2,25]. The results of this experiment begin to suggest the noradrenergic system does not demonstrate a similar effect as dopamine on semantic processing, at least with the drugs and doses used in this study. Our results did show a direct and indirect priming effect on placebo which has been well-established in the literaure [6,25,30], but drugs increasing noradrenergic activity did not appear to inhibit the priming effect for indirectly related words as occurs with dopamine. The only trend detected was towards there being a drug effect on closely related pairs, with a trend towards a faster response on ephedrine compared to placebo for closely related word pairs. This is the opposite of what would be expected if noradrenergic agonists had an inhibitoty effect on semantic priming as occurs with dopaminergic agonists. Therefore, in contrast to dopamine, which restricts the semantic network to more closely related nodes, it seems that norepinephrine does not have such an effect as assessed by this task, or the effect is not as potent with the drugs and doses used in this study.

This data supports the hypothesis that the restriction of the semantic network seen with dopaminergic modulation does not occur with noradrenergic agents, although due to the small sample size this conclusion must be considered preliminary. This may imply that the effects of dopaminergic agents on semantic priming are not due to conversion to norepinephrine. Further study would be necessary to more directly address this implication. The effects of D1 agonists on priming does, though, also provides further support for this hypotheses [37]. The findings also contrast with the previous body of work showing that the noradrenergic system affects lexical/semantic network flexibility during anagram solving [7,8], and in the setting of stress and performance anxiety [1,20]. In the context of these other findings [1,7,8,20,37,40], it seems that dopamine affects the lexical/semantic network on more automatic processing such as word recognition tasks, whereas norepinephrine restricts the network for more controlled searches of the lexicon. However, there are several limitations to this study. First the hemodynamic effect of ephedrine did not reach significance in this study. Whereas the hemodynamic effect of ephedrine in our previous work at the same dose has been marginal [7,14], the lack a significant effect in the present study may have impacted our results. Previous research, though, has revealed a number of significant cognitive effects with this exact dose of ephedrine [7, 29]. Second, whereas ephedrine affects multiple alpha and beta adrenoreceptor types and also has some dopaminergic and serotonergic effects, propranolol is beta-selective, which may affect the results. Future work will be necessary to further investigate this.

Whereas dopaminergic agents do impact priming [2, 25], recent evidence suggests that there is no dopaminergic effect in studies on more controlled lexical searches such as verbal problem solving [40]. The current study suggests that whereas noradrenergic agents impact controlled lexical searches [9], there appears to be no such effect observed with priming. Further work examining effects of noradrenergic and dopaminergic agents on other controlled and automatic processes as well as explicit and implicit tasks is necessary to better understand these effects. In addition to the priming effects, research in animal models has shown varying effects of dopaminergic agents on set-shifitng tasks [22]. In particular, agonists of both D1 and D2 had no effect on set-shifting whereas D2 antagonists impaired set-shifting in rodents [22]. NMDA receptors in the prefrontal cortex and the nucleus accumbens core also interact to affect set shifting [21,42]. D2 antagonists have also been shown to impair set-shifting in human volunteers [31]. A computational model has been proposed wherein phasic stimulation of D2 receptors in the striatum drives flexible adaptation of cognitive representations maintained by the prefrontal cortex [15]. Dopamine also has demonstrated effects on working memory [28]. As with the dopaminergic system, receptor selectivity is observed with noradrenergic effects on cognition. Norepinephrine is important in arousal [17,18,39]. A range of other cognitive effects have also been noted with noradrenergic agents, including effects on motor learning [23], response inhibition [12], working memory and emotional memory [13]. Alpha-1 adrenergic activity affects attentional set shifting [27], and alpha-2 adrenergic activity affects working memory [3,4]. As described above, decreased beta-adrenergic activity appears to be associated with improved performance in tasks requiring a high degree of flexibility of access to the lexical/semantic network [1,7–9], whereas models suggest that increased noradrenergic activity is associated with increased switching on a two alternative forced choice task [5,44]. Whereas preliminary, the evidence from the present study, at least when taken in context of findings of previous work [1,7,8,20,37, 40], suggests that regarding effects on the semantic network, the dopaminergic system appears to affect the semantic network for priming but not verbal problem solving, and the noradrenergic system affects the lexical/semantic network for verbal problem solving but not priming. Future work will be needed to examine the effects of interactions between these neurotransmitter systems, and to further clarify our understanding of the underlying neuroanatomical substrate of these effects.

## Acknowledgements

This study was supported by NIDA (DA15734) (Beversdorf) and NINDS (NS043222) (Beversdorf). Preliminary results of this research were presented in abstract form at the American Academy of Neurology, 2004.
